# Supported ITZ Modification Efficiencies via Surface Coating Nanoparticles on Aggregate and its Influence on Properties

**DOI:** 10.3390/ma12213541

**Published:** 2019-10-29

**Authors:** Kai Wu, Hao Han, Linglin Xu, Xiaojie Yang, Geert De Schutter

**Affiliations:** 1 Key Laboratory of Advanced Civil Engineering Materials, Tongji University, Ministry of Education, Shanghai 201804, China; wukai@tongji.edu.cn (K.W.); 15300952706@163.com (H.H.); xulinglin@126.com (L.X.); 2 School of Materials Science and Engineering, Tongji University, Shanghai 201804, China; 3 Magnel Laboratory for Concrete Research, Ghent University, 9052 Ghent, Belgium; geert.deschutter@ugent.be

**Keywords:** interfacial transition zone, coating, nanoparticles, microstructure, quantitative image analysis

## Abstract

In order to modify the porous interfacial transition zone (ITZ) microstructure of concrete more efficiently, a method of coating aggregate surfaces by using several nanoparticles was evaluated in this study. The compressive strength, chloride penetration of sound, and pre-loading samples were assessed in relation to the type of coating materials used (slag, nano-CaCO_3_, and nano-SiO_2_) and the designed coating thickness (5, 10, and 15 μm). The ITZ microstructure was quantitatively determined via Backscattered electron (BSE) image analysis. Results showed that the overall performance of concrete is highly dependent on the coating materials and the designed coating thickness. Increasing the coating thickness of slag and nano-SiO_2_ could improve the chloride penetration resistance but decrease the compressive strength. Using nano-CaCO_3_ to coat the aggregate leads to a significant reduction in the properties of the so-prepared concrete. Though coating inert fine particles around aggregate could disturb the initial particle packing and modify the ITZ, it is not able to improve the overall concrete properties. Coating aggregate could determine the ITZ microstructure, especially within the region that is around 30 μm away from aggregate surface.

## 1. Introduction

Concrete is by far the most common building materials because of its low cost, the abundance of raw materials required, and its adaptability and versatility in manufacturing various structural shapes and environments. Investigations into the properties of concrete normally treat it as a whole, but concrete is fundamentally a composite material, having hydraulic paste diluted with coarse and fine aggregates with an interfacial transition zone (ITZ) between them. The properties of aggregate and bulk cement paste have been investigated deeply and extensively [1], but there is still uncertainty regarding the influence of the ITZ on the overall performance of concrete. Due to a lack of understanding about this weak link in concrete, a few proposals have been put forward to modify it [2,3,4]. Moreover, there is no simple method of predicting how much improvement can be made in the overall performance through enhancing the ITZ.

The wall effect has normally been used as the origin for the formation of the ITZ. The difference in size between aggregates and cement powder means that each aggregate can be considered as a wall that disrupts the packing of the cement particles and results in a region close to the aggregate that contains predominately small particles and has a relatively higher porosity [5]. Quantitative image analysis confirms that the ITZ is characterized by the disruption packing of anhydrous [6]. Considering the fresh state of the mixture, the anhydrous becomes loose in the region close to the aggregates. Micro-bleeding leads to an accumulation of water under the aggregate, and the fresh paste or mortar exhibits a phenomenon of water and anhydrous cement gradient distribution [7]. Finally, the porosity increases from the bulk cement matrix to the aggregate surface.

Considering the origination, the modelled ITZ has gradients extending out from the aggregate to the bulk cement paste. Combining the information from the literature [5,8,9,10], the microstructure of the ITZ can be summarized as follows: (i) A duplex film of Ca(OH)_2_ topped by or occasionally intermixed with Calcium Silicate Hydroxide (CSH) located in the vicinity of the aggregate. This film could transform to a dense layer, followed by a deposit of CSH in the form of fiber; (ii) the region enriched by large Ca(OH)_2_ crystals and ettringite (AFt) is located after the duplex layer. Moreover, another feature of the ITZ is that a relatively higher porosity could be observed due to the orientation growth of calcium hydroxide and ettringite crystals in an open space.

According to these features, the ITZ microstructure can be modified by changing the particle arrangement, consuming the calcium hydroxide and reducing the micro-bleeding [7,11,12,13]. The published results have demonstrated that using blended materials can cause a significant reduction in ITZ thickness and porosity, thus improving performance related to mechanical durability [2,14,15,16,17]. The mechanism includes the micro-filler effect contributed by the finer particles, and the nucleation site effect from the precipitation of fine hydration products and the reduction of the preferred orientation. The latent hydration and pozzolanic reaction might reduce the content of calcium hydroxide (CH) within the ITZ as well [3,4,18,19]. This function can also be found during the application of nanomaterials. Although great achievements have been made using blended materials to modify the ITZ, less work has been done regarding the nanoscale features of the materials.

Due to the multi-scale features of concrete microstructure, it has been well accepted that the utilization of nanomaterials can enhance the overall performance [20,21,22]. When incorporated into the concrete, the nanoscale features of the materials would exert a significant effect on the hydration of the cement, and thus its mechanical properties and durability [23,24,25,26]. The improvements are normally ascribed to the hydration seed effect, micro-filler effect, and pozzolanic reaction [20,27]. Therefore, it can be inferred that the application of nanomaterials also has the potential to change the ITZ microstructure. The work reported in the literature is normally performed on neat cement paste or concrete. The information regarding the effect of nanomaterials on the ITZ in relation to the overall performance is still limited. Therefore, this work was designed to evaluate the possible effect of ITZ modification on the overall performance. A method of coating the aggregate surface by using several types of nanomaterials was applied. The ITZ microstructure was determined through BSE image analysis, quantitatively. The mechanical properties and the chloride penetration before and after pre-loading of the so-prepared specimens were evaluated in relation to the types and dosages of nanomaterials.

## 2. Experimental

### 2.1. Materials

**Figure 1:**
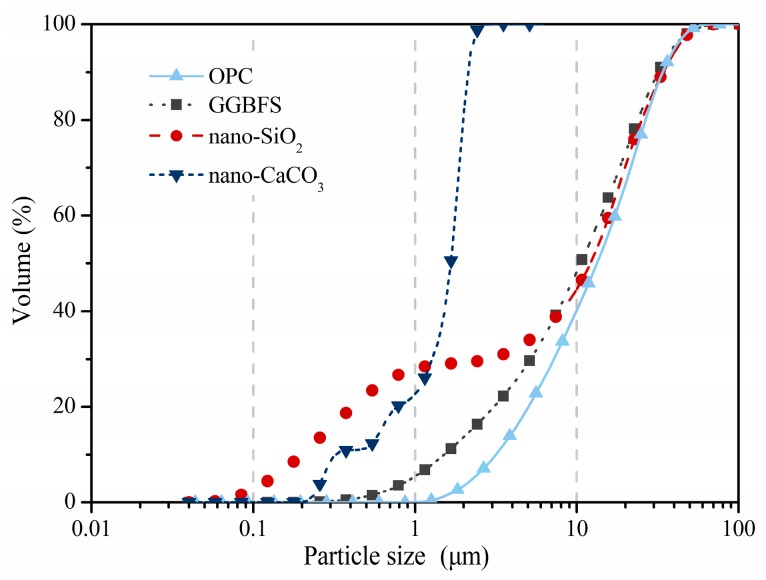
Particle size distribution of the applied materials.

**Figure 2:**
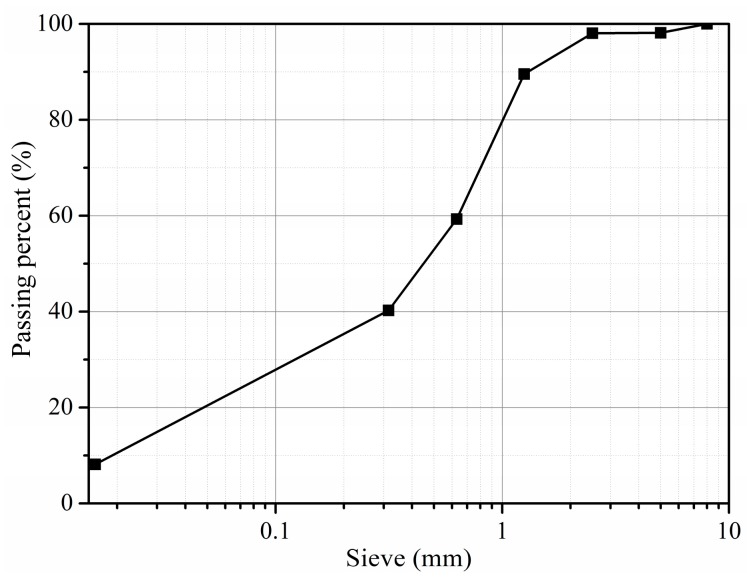
Sieve analysis of the used aggregate.

The Portland cement (Conch Group, Wuhu, China) used in this work was grade 52.5 (OPC). Two types of nanomaterials, according to their reactivity, i.e., nano-CaCO_3_ (n-C) and nano-SiO_2_ (n-S) were applied to the aggregate surface. Since it has been proved that the addition of slag is able to modify the ITZ [28,29], ground granulated blast furnace slag (S) was employed as a coating material for reference. The chemical compositions and physical features of the employed materials are given in Table 1. Since the fineness is one of the most important parameters for particle packing within the ITZ, the size distributions were measured using laser diffraction (Mastersizer 2000, Malvern Panalytical Ltd., Malvern, UK), and the results are shown in Figure 1. The sand and gravels were mixed together, and the sieve analysis of the final aggregate is shown Figure 2.

### 2.2. Mixture

The powders were designed to coat aggregate, and the dosage of slag, n-C, and n-S were calculated using a volume method. According to the grain size and distribution of the aggregate, an approximate coated thickness of 5, 10, and 15 μm was designed, and the final volume dosage, calculated from the “void exclusion probability” for each thickness, was 1.47%, 2.97%, and 4.52%, respectively. The detailed determination process can be found elsewhere [30,31]. In order to avoid the influence of aggregates on each other, the total aggregate volume content in each mixture was set at 45%. After weighting all the solids, the fresh mixtures were prepared with a water to binder (w/b) ratio of 0.35, and appropriate amounts of superplasticizer (SP) were added to control the flow value of the fresh mixture at 150 mm and to avoid bleeding [32]. The detail of compositions for the mixture are given in Table 2.

### 2.3. Sample Preparation

**Figure 3:**
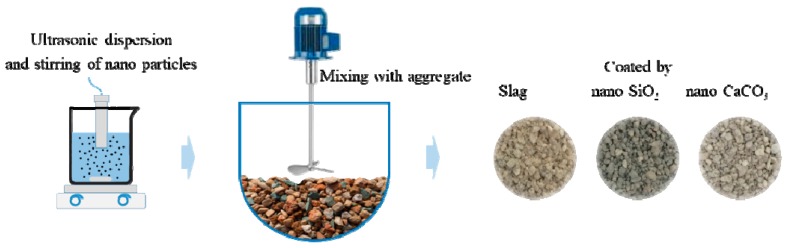
Schematic of aggregate coating process.

In order to distinguish the ITZ from the bulk cement paste, the nanoparticles were initially coated on the aggregate before mixing. After weighting, the slag, n-C, and n-S, were dispersed in ethanol with ultrasonic dispersion and stirring for 15 min. The so-prepared slurry was than mixed with aggregate with a rotation speed of 30 r/min for 3 min. The coated aggregate was transferred to an oven at 45 ± 5 °C for 1 h. The schematic flow of the preparing procedure is shown in Figure 3. The so-prepared aggregate was stored for concrete mixture preparation.

All mixtures were cast into 40 × 40 × 160 mm^3^ prisms for a compressive strength test, and 150 × 150 × 150 mm^3^ cubes for chloride migration measurement. The fresh mixtures were vibrated until no more air bubbles were released. All the specimens were demolded after 24 h and then stored in the curing room at 20 ± 2 °C and relative humidity (R.H) 95% ± 5% until the designated age. After 28 days, three cylinders with a diameter of 100 mm and a height of 50 mm were drilled and cut from each cube for the chloride migration test.

### 2.4. Mechanical and Transport Properties

According to the procedure of the Chinese standard GB/T 17671, compressive strength tests were performed on the specimens after breaking the prisms in two at the age of 3, 28, and 56 days. The loading speed was set at 2400 ± 200 N/s by using DY-208 Auto-press (Jianyi experiment instrument, Wuxi, China). The maximum compressive force of the machine is 300 kN. The final results represented are the average values obtained on six prisms.

After 56 days of curing, the non-steady-state chloride migration test was carried out on the drilled cylinders [33]. Before testing, the so-prepared cylinders (*Φ*100 × 50 mm^2^) were vacuum saturated with Ca(OH)_2_ solution. Thereafter, a direct current (DC) electrical potential was applied on the specimens to force the chlorides (in 10% NaCl solution) into the concrete. After migration, the specimens were split in two and sprayed with 0.1 mol/L AgNO_3_. The penetration depth was measured for the determination of migration coefficients following Equations (1)–(3):

(1) $$D_{m}=\frac{RT}{zFE}\frac{x_{d}-\alpha \sqrt{x_{d}}}{t}$$

(2) $$E=\frac{U-2}{L}$$

(3) $$\alpha =2\sqrt{\frac{RT}{zFE}}erf^{-1}(1-\frac{2c_{d}}{c_{0}})$$

where *D_m_* is the chloride migration coefficient (m^2^/s), *U* is the absolute applied voltage (V), *R* is the gas constant (8.314 J/(K∙mol)), *z* is the absolute ion valence, *F* is the faraday constant (9.648 × 10^4^ J/(V∙mol)), *T* is the average temperatures in the anolyte solution before and after tests (K), *L* is the specimen thickness (m), *x_d_* is the average chloride penetration depth (m), *t* is the experimental duration (s), *c*_0_ is the chloride concentration in the catholyte solution, and *c_d_* is the chloride concentration at which the color changes (0.07 mol/dm^3^).

The ITZ has been considered as the weakest region since normal concrete fails at a considerably lower strength level than that of either bulk cement paste or aggregate. It has been indicated in the literature that the interfacial bond was the dominant factor for the bending resistance and played an important role on the compressive strength [34,35,36]. The presence of cleavable calcium hydroxide and the higher porosity could promote the growth of microcracks [37]. In order to assess the modification efficiency of coating nanoparticles on aggregate, two groups of cylinders were prepared and loaded until reaching 60% and 80% of the ultimate compressive strength obtained before. The loading pressure was kept for 10 min. After pre-loading, chloride migration tests were performed on the cylinders.

### 2.5. ITZ Microstructure Determination

One of the most remarkable features of ITZ is its high porosity, therefore, a quantitative SEM (FEI Company, Hillsboro, OR, USA) method using the backscatter model was applied to study the effect of aggregate coating on the ITZ microstructure. The process involved imaging and data processing. To avoid the surface effect, slices (40 × 40 × 10 mm^3^) were cut from the inner part of the concrete samples (150 × 150 × 150 mm^3^) by using a lime-water lubricated diamond-bladed wheel at 56 days. Prior to grinding and polishing, the obtained slices were submerged in liquid nitrogen for 5 min to stop further hydration. After freezing, the slices were moved into a freeze-dryer with −40 °C and 0.1 Pa for two weeks. For the BSE observations, the slices were vacuum penetrated with a low viscosity epoxy resin and dried at 40°C for one day. After hardening, the samples were ground with 320, 500, 1200, and 2400 grit SiC paper for 4 min each, and polished with diamond paste of 3, 1, and 0.25 μm for 2 min each. The final polished samples were cleaned up with a low-relief polishing cloth. The polished samples were examined under the BSE detector in an environmental scanning electron microscope. The magnification used for evaluation was 500×, and the pixel resolution was 1024 × 943. Around 45 images were captured for each sample and used for image analysis.

**Figure 4:**
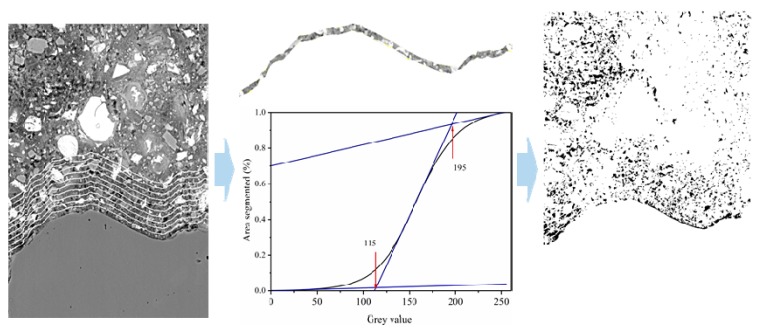
Schematic of BSE imaging process including strip delineation, segmentation, and porosity determination.

In this study, a “concentric expansion” method was employed to delineated strips with a certain thickness from the aggregate surface. The detailed procedure can be found in the previous study [29,38], and the schematic flow of the imaging process is shown in Figure 4. The final results are presented by plotting the porosity of each strip against the distance away from the aggregate surface, and thus the effect of nanoparticles on ITZ microstructure can be assessed.

## 3. Results and Discussion

### 3.1. Compressive Strength

**Figure 5:**
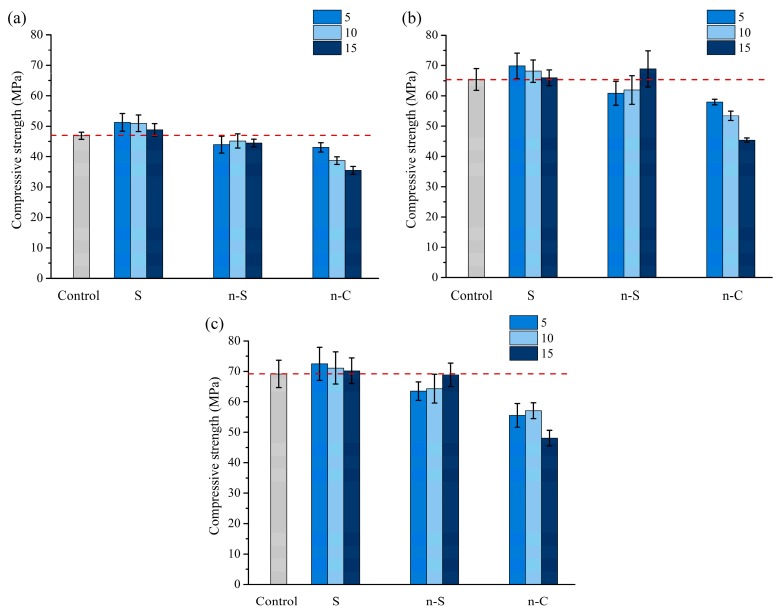
Effect of coating particles and thickness on the compressive strength: (**a**) 3 days; (**b**) 28 days; (**c**) 56 days.

The ITZ has normally been considered as the weakest region in concrete, and, therefore, exerts a greater influence on the mechanical properties than can be expected from its size [39,40,41]. The ITZ serves as a bridge between aggregates and cement paste. Even when the individual components are of high strength, the mechanical properties of the concrete may be weakened due to the bridge’s poor effect that does not allow stress transfer. The compressive strengths of various mixtures in relation to curing age is given in Figure 5. It can be observed that the effect of coating materials on the compressive strengths is highly dependent on the type of nanoparticles and testing age. The coating of slag is able to improve the overall strength in comparison with the reference. When the designed coating thickness is increased, the compressive strength decreases slightly. Regarding the nanoparticles, coating nano-SiO_2_ around aggregate tends to reduce the compressive strength until 15 μm of thickness, i.e., 4.52% dosage by volume was applied. However, the coating of nano-CaCO_3_ reduces the compressive strength regardless of the curing age. This reduction is more significant with higher nano-CaCO_3_ dosage.

It is believed that the ITZ microstructure is initially determined by the particle packing due to the size difference between cement grains and aggregate particles. The well known “wall effect” leads to a depletion of larger anhydrous cement grains, while there is a relatively high amount of smaller ones and a high w/c ratio [7,42]. The use of nanoparticles could densify the initial porous region via micro-filling. It seems that the densification could not increase compressive strength in the groups of nano-CaCO_3_. The presence of nano-CaCO_3_ can increase the effective water to cement ratio, which could result in a more porous structure. On the other hand, the nanoparticles could work as nucleation sites for the deposition of crystals and promote some of the calcium hydroxide crystals to react with the remaining aluminates to form hemi-carbonate. The particle size of nano-SiO_2_ is coarser than n-C. The coating of n-S could consume parts of the calcium hydroxide through pozzolanic reaction in spite of the nucleation and micro-filler effect. This modification can only be observed until 15 μm thickness were coated. The application of slag as a coating material is the most effective way to improve the compressive strength. Although the mean particle size of slag is close to that of cement, it is still expected to have a microfiller effect because the proportion of finer particles is higher in slag, as can be seen in Figure 1. Moreover, the hydration of slag is able to further densify the ITZ and increase the compressive strength. Generally, only using finer particles, such as nano-CaCO_3_, to fill the porous region is not enough to modify the mechanical strength.

### 3.2. Chloride Migration

**Figure 6:**
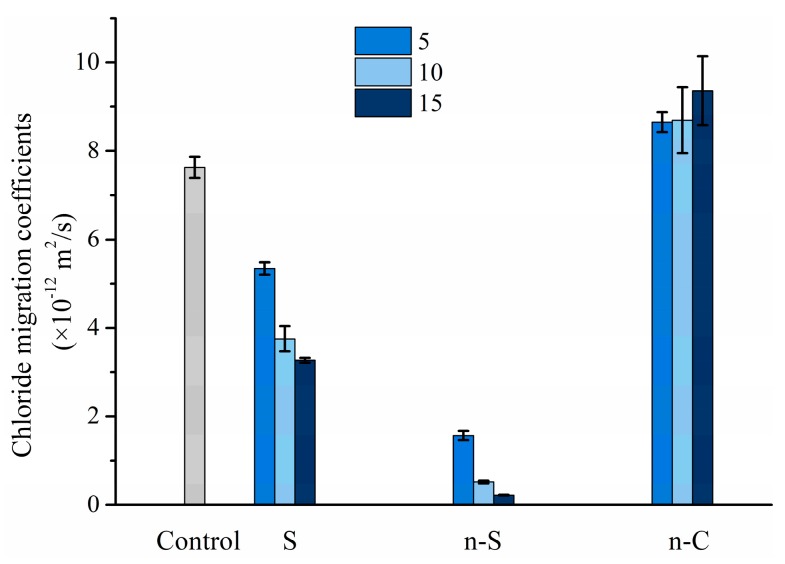
Effect of coating particles on the chloride migration of specimens after 56 days of curing.

The effect of coating materials on the chloride migration of specimens after 56 days of curing is shown in Figure 6. Similar to direct blending in the mixture, the coating of slag on aggregate in advance is also able to limit the penetration of chlorides. In this study, the highest dosage of slag is S15, which corresponds to 17.25% of cement by mass. It seems that coating slag on the aggregate is less effective in resisting chloride migration in comparison to blending it directly into the mixture [29]. In the previous study, the improvement in chloride penetration resistance by using slag could be partially responsible for the improvement of the ITZ, but more importantly, to the densification of bulk cement paste. Unlike the trend observed in compressive strength measurements, the pre-coating of n-S is able to decrease the chloride migration coefficient significantly, and is more effective than the S group. This reduction is more significant with the increase of n-S coating thickness.

Regarding the n-C group, a similar trend is observed, as can be seen in the compressive strength measurement. The coating of CaCO_3_ would promote the penetration of chlorides in comparison with the reference. The chloride migration coefficient increases with the designed nano-CaCO_3_ coating thickness. On the one hand, the coating of CaCO_3_ can only vary the ITZ by the filling effect, while the slag and SiO_2_ can consume most of the calcium hydroxide. On the other hand, a portion of the coated particles would release and merge into bulk cement paste during mixing, which would further weaken the efficiency.

### 3.3. Chloride Migration after Pre-Loading

**Figure 7:**
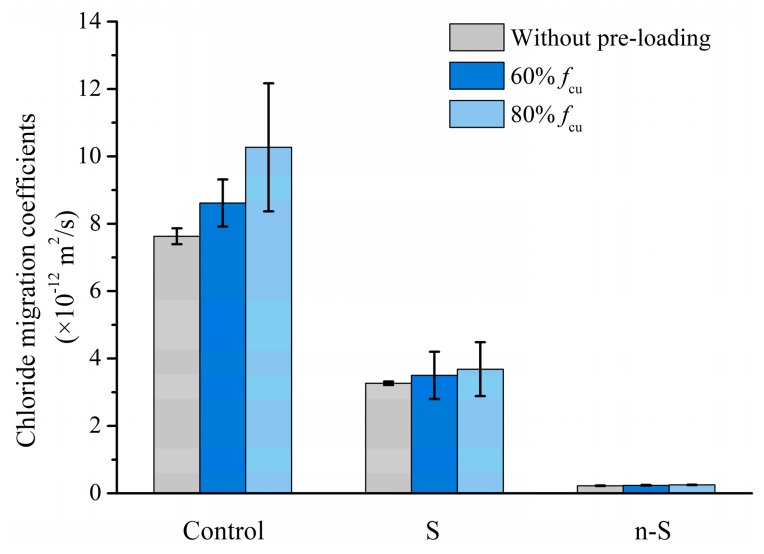
Chloride migration coefficient of specimens after being pre-loaded.

The chloride migration coefficient of specimens after pre-loading before reaching 60% and 80% of ultimate compressive strength is shown in Figure 7. The coefficients are also compared with the result of corresponding sound specimens. With respect to the reference group, the chloride migration coefficient increases by around 40% after 80% of ultimate compressive strength was applied on the specimens for 10 min. However, the pre-loading does not seem to promote the chloride migration in the S and n-S group, even after 80% of the ultimate compressive strength was applied.

Concrete fails at a remarkably lower strength level than the stiffness of either the hardened cement paste or the aggregate. Microcracks tend to be formed and developed at the ITZ under loading. The ease of microcrack propagation within the ITZ is dominated by the difference in strain mismatch between the aggregate and the bulk cement paste under a given loading [43]. The slight difference in the chloride migration coefficient between the sound and preloading specimens in the S and the n-S group indicates that coating slag and nano-SiO_2_ around aggregate could modify the ITZ.

### 3.4. The ITZ Microstructure

#### 3.4.1. Morphology

**Figure 8:**
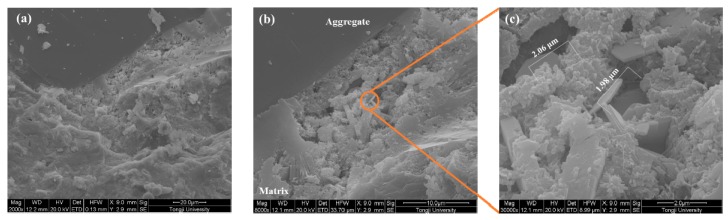
Microstructure of the interfacial transition zone (ITZ) of the control sample. (**a**) 2000×; (**b**) 8000×; (**c**) 30000×.

**Figure 9:**
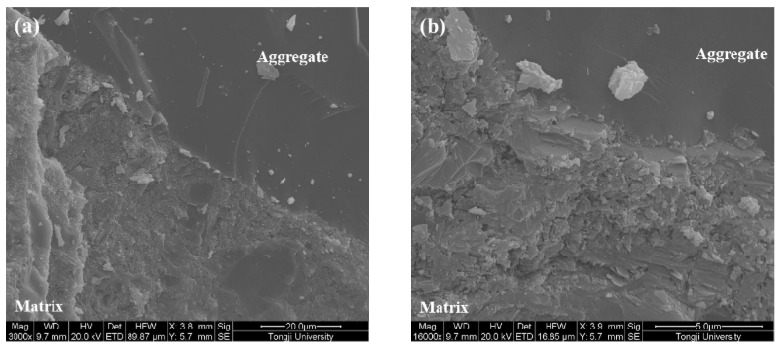
Microstructure of the ITZ of the slag coated aggregate sample. (**a**) 3000×; (**b**) 16000×.

**Figure 10:**
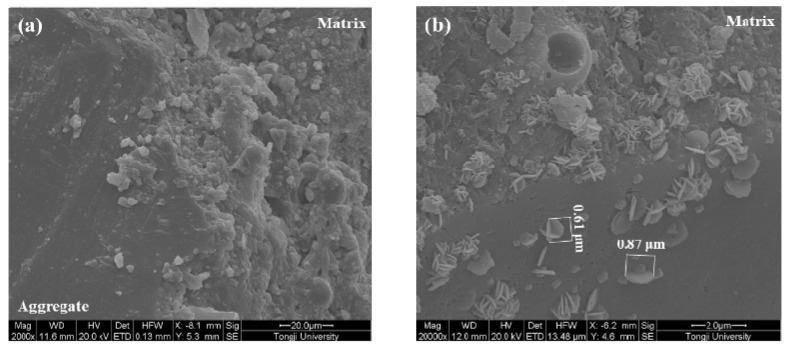
Microstructure of the ITZ of the nano-SiO_2_ coated aggregate sample. (**a**) 2000×; (**b**) 20000×.

**Figure 11:**
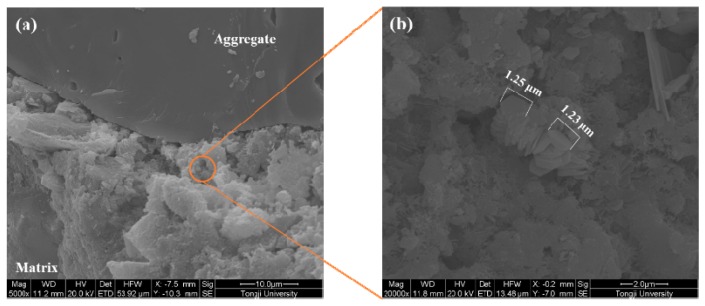
Microstructure of the ITZ of the nano-CaCO_3_ coated aggregate sample. (**a**) 5000×; (**b**) 20000×.

The microstructure of the ITZ of samples made with various coating materials is shown in Figure 8, Figure 9, Figure 10 and Figure 11. A typical ITZ microstructure is usually characterized by a contact layer that is adjacent to the aggregate surface and essentially composed of Ca(OH)_2_ covering a network of ettringite. The contact layer is adjacent to the intermediate zone and consists of leaf- or flak-like Ca(OH)_2_, needle-shaped ettringite, and sporadic needle-shaped CSH. The dense part in the ITZ would merge into bulk cement paste [44]. Regarding the control sample, as shown in Figure 8, a typical ITZ microstructure can be observed, which has a relatively higher porosity compared with the morphology in the matrix. The precipitation of Ca(OH)_2_ can be easily found in the pores. Although large pores can be observed, there is still some dense areas located in the ITZ, confirming its highly heterogeneous feature.

In comparison with the control sample, a denser ITZ can be easily found in the specimens containing aggregate pre-coated by slag, even at a higher magnification (Figure 9). The bulk cement paste is in close contact with the aggregate surface. No porous region can be observed in the ITZ. Therefore, the samples made with slag pre-coated aggregate show a reduction of the chloride migration coefficient and an improvement in compressive strength. A similar phenomenon is also found in the sample made with nano-SiO_2_ pre-coated aggregate, as can be seen in Figure 10a. However, some large particles locate close to the aggregate due to the agglomeration of fine nano-SiO_2_. The agglomerated particles are not consumed via pozzolanic reaction even after 56 days of hydration. This could be the reason why the coating of nano-SiO_2_ around aggregate did not improve the compressive strength as significantly as it did with slag. It is still expected that part of the nano-SiO_2_ reacts with the Ca(OH)_2_ and modifies the pore structure by refining the pore size, which contributes to the improvement of chloride migration resistance. Moreover, the size of the precipitated Ca(OH)_2_ on the aggregate surface (Figure 10b) is much finer than that observed in the control samples due to the nucleation site effect. The SEM images of the samples prepared with nano-CaCO_3_ pre-coated aggregate in Figure 11 show a more porous microstructure in comparison to the other two coating materials. This further confirms the reduction of chloride penetration resistance and the compressive strength of the so-prepared specimens. Although it is suggested that the nano-CaCO_3_ is inert, it is still able to be the nucleation site for the crystallization of finer Ca(OH)_2_, as shown in Figure 11b.

#### 3.4.2. Porosity Distribution

**Figure 12:**
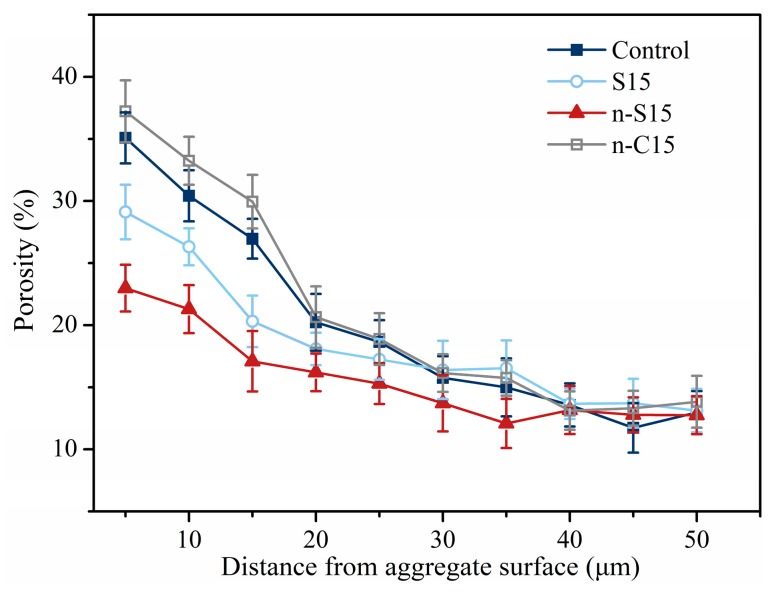
Effect of coating particles on the porosity distribution within the ITZ.

The porosity profiles in relation to the type of coating materials are shown in Figure 12. It can be seen that the porosity reduces with the distance away from the aggregate surface, which is in line with the expected trend of a typical ITZ microstructure. Coating slag and nano-SiO_2_ on the aggregate in advance leads to a reduction in the porosity close to the interface. The reduction is more remarkable for the sample prepared with nano-SiO_2_ pre-coated aggregate, which is different from the findings in the compressive strength and chloride migration tests. This could be due to the higher reactive nature of SiO_2_ in comparison with slag. Although part of the nano-SiO_2_ was not consumed, the occurred pozzolanic reaction is still able to modify pore structure from coarse sizes to finer ones. The pixel resolution for each image is 1024 × 943, and the ultimate resolution is approximately 0.46 μm per pixel. Therefore, the fine pores that were mainly caused by the pozzolanic reaction can be ignored during image analysis, and the porosity of n-S15 is lower than that of S15. The highest porosity is observed in samples made with nano-CaCO_3_ pre-coated aggregate, which is in agreement with the findings in the overall performance experiments. Moreover, there is no significant difference in porosity between the four mixtures beyond 30 μm distance from the aggregate surface, and the porosity reduces to around 15%, which is also close to the values of bulk cement paste at a distance of 50 μm. It suggests that coating particles around aggregate determines the ITZ microstructure; in this work, the influence distance is around 30 μm.

## 4. Conclusions

The ITZ between the aggregate and the bulk cement paste is characterized by a relatively higher porosity and larger pore size and orientation deposition of large Ca(OH)_2_ crystals, and is normally considered as the weakest region in concrete. In this paper, a method of surface coating nanoparticles on aggregate was applied to modify the ITZ microstructure. A quantitative image analysis was employed to determine the porosity distribution within the ITZ. The impact of the so-prepared aggregate on the compressive strength, the chloride migration of sound, and pre-loaded specimens were also evaluated.

Coating slag and nano-SiO_2_ on aggregate improves the compressive strength and the chloride penetration resistance. The improvement is dependent on the type of coating materials and the designed coating thickness. Increasing the designed coating thickness of slag and nano-SiO_2_ can limit the chloride penetration but decreases the compressive strength. Although a significant reduction in chloride penetration resistance and compressive strength is observed in samples with a nano-CaCO_3_ coating, it is still expected to densify the ITZ through micro-filling. The reduction could be due to the grain release and the merge into bulk cement matrix during mixing.

A much denser ITZ can be found in the specimens made with aggregate pre-coated by slag, even at a higher magnification. The bulk cement paste is in close contact with the aggregate surface. The ITZ microstructure of samples prepared with nano-SiO_2_ coated aggregate is also densified, but some agglomerated particles can still be observed in the micrograph and limit the modification efficiency. Coating aggregate with nano-CaCO_3_ promotes the deposition of fine Ca(OH)_2_ crystals. The BSE image analysis clearly confirms the modification of the ITZ microstructure by coating slag and nano-SiO_2_ around the aggregate in advance. Coating particles could influences the microstructure of the region that is less than 30 μm away from the aggregate surface, mainly located in the ITZ.

## Figures and Tables

**Table 1:** Chemical compositions and physical features of materials.

Materials	Compositions (wt.%)							Specific Surface Area (m^2^/g)	Density (kg/m^3^)
	CaO	Al_2_O_3_	SiO_2_	MgO	Fe_2_O_3_	SO_3_	K_2_O		
OPC	63.12	4.94	18.73	1.02	3.99	3.07	-	397	3163
Slag	39.50	14.91	34.40	5.84	0.39	-	0.33	450	2652
n-C	55.6	-	-	-	-	-	-	26000	2557
n-C	-	-	99.1	-	-	-	-	23000	2253

**Table 2:** Mix design and final proportions.

No.	Nanoparticles Volume (%)	Nanoparticles (kg/m^3^)	Aggregate Volume (%)	Cement (kg/m^3^)	Aggregate (kg/m^3^)	Water (kg/m^3^)	SP (wt.%)
Control	0	0.0	45	825.6	1184.9	289.0	0.037
S05	1.47	39.0		783.1	1184.9	287.7	0.035
S10	2.97	78.8		739.7	1184.9	286.4	0.032
S15	4.52	119.9		694.8	1184.9	285.1	0.032
n-S05	1.47	33.1		786.2	1184.9	286.7	0.040
n-S10	2.97	66.9		745.9	1184.9	284.5	0.044
n-S15	4.52	101.8		704.3	1184.9	282.1	0.046
n-C05	1.47	37.6		783.8	1184.9	287.5	0.054
n-C10	2.97	75.9		741.1	1184.9	286.0	0.055
n-C15	4.52	115.6		697.1	1184.9	284.4	0.061
